# Families Living with Blood-Borne Viruses: The Case for Extending the Concept of “Serodiscordance”

**DOI:** 10.1155/2017/4352783

**Published:** 2017-10-18

**Authors:** Asha Persson, Christy E. Newman, Myra Hamilton, Joanne Bryant, Jack Wallace, kylie valentine

**Affiliations:** ^1^Centre for Social Research in Health, Goodsell Building, Level 3, UNSW, Sydney, NSW 2052, Australia; ^2^Social Policy Research Centre, Goodsell Building, Level 3, UNSW, Sydney, NSW 2052, Australia; ^3^Australian Research Centre in Sex, Health & Society, La Trobe University, 215 Franklin Street, Melbourne, VIC 3000, Australia

## Abstract

The concept of “serodiscordance” (mixed infection status) is primarily associated with epidemiological concerns about HIV transmission risk in couples. We make the case for extending this concept to include families with mixed HIV and viral hepatitis status. Social research on couples with mixed HIV and hepatitis C status has laid an important foundation for illuminating how experiences of serodiscordance within intimate partnerships are much broader than concerns about risk. This body of work attests to serodiscordance holding promise as a valuable concept for understanding viral infections as socially situated and intensely relational phenomena. However, serodiscordance is still limited as a concept because of its near universal focus on couples. It is rarely applied to wider relationships, including family networks beyond the couple. Despite evidence in the literature that families are affected by blood-borne viruses in multiple social, emotional, financial, and generational ways, the concept of serodiscordance does not capture these broader dynamics. Making serodiscordance more inclusive is an important step in recognising the diverse ways families' everyday lives, relationships, and futures can be entangled with HIV, hepatitis C, and hepatitis B, and for understanding how today's era of effective treatment options might shape the “family life” of viral infections.

## 1. Introduction

In the contemporary “treatment era,” blood-borne viruses are undergoing profound changes. New biomedical technologies to prevent HIV infection have enlivened hopes of ending the global epidemic [1] and are transforming social and sexual relations due to the significantly reduced risk of transmission and the possibility of conception without clinical interventions [2–4]. The recent introduction of highly effective treatments for hepatitis C, which are also far less arduous than earlier treatment, is setting up radical expectations that the virus can be globally eliminated by 2030 [5]. Similar ambitions for hepatitis B have recognised the need to scale up screening, treatment, and childhood vaccination to help prevent the kind of intergenerational transmission of the virus that has led to significant morbidity and mortality among many populations [5].

In this article, we make the case for extending the concept of “serodiscordance” in the context of this current treatment moment, with all of the associated promises, possibilities, and unknown knock-on effects it brings. “Serodiscordance” is widely used in the HIV literature to describe a relationship between two people with different viral statuses, in other words a person with a virus and another person without it. It is most commonly associated with HIV, but it does appear in the hepatitis C literature, though not in the hepatitis B literature. The concept of serodiscordance has been crucial in beginning to shift the emphasis beyond individuals in the study of blood-borne viruses. Yet, it is still constrained as a concept because of its near universal focus on couples and transmission risk. While the existing literature suggests that a viral infection can impact on and involve families (and broader social networks) in multiple ways, and not only individuals, the concept of serodiscordance as it is normatively used and understood does not capture these socioemotional and familial dynamics.

Lifting our gaze from the preoccupation with “risk” in relation to these blood-borne viruses is vital to understanding how today's era of effective treatment options might shape the “family life” of viral infections. Recently, we have seen attempts to broaden serodiscordance beyond the focus on risk to gain important insights into the social and emotional dimensions of hepatitis C and HIV among mixed-status couples. However, we still lack a concept for understanding blood-borne viruses in the wider family contexts in which they are often situated and experienced. There is no objective reason why the concept of serodiscordance could not be extended to mixed-status families (and beyond) given it simply means differing* (discordant)* blood* (sero)*. Making serodiscordance more inclusive in this manner is an important step in recognising and drawing much needed attention to the diverse ways families' everyday lives, relationships, and futures can be entangled with these viral infections.

## 2. Relationality and Serodiscordance

The notion that illnesses are profoundly relational [6, 7] is clearly borne out by viral infections such as HIV, hepatitis B, and hepatitis C. Not only are they relational in how they are acquired, transmitted, and prevented, but also because they are inexorably enacted, perceived, and managed through a range of socially situated relationships, meanings, and practices. As with other chronic illnesses, these blood-borne viruses can have broad impacts that extend far beyond affected people, shaping the everyday experiences and emotional lives of their relational networks, including their families. And yet, historically, individuals and their behaviours have been the primary focus in public health research and interventions, due to a complex mix of epistemological hegemony, methodological criteria, funding priorities, and the politics of “evidence” [8–11]. In regard to HIV, Rotheram-Borus and colleagues [12] also speculate that the lack of attention to the relational context of families is a legacy of the epidemic's emergence in the USA with its strong cultural emphasis on individualism, and the fact that HIV initially affected mainly gay men who were perceived as disconnected from traditional family structures. Seear and colleagues [13] suggest that a similar individualising tendency in hepatitis C research likely stems from the virus' association with injecting, with people who inject drugs typically cast as lacking any capacity for intimate relationships. In relation to hepatitis B, research is essentially focused on biomedical descriptions of the virus and, to a far lesser degree, its social impact on individuals, with an absence of explorations of its impact on family life, despite the intrafamilial nature of the virus.

As these epidemics have unfolded across the world and across populations, this individual focus is proving highly inadequate for understanding the real-life contexts of blood-borne viruses. People with these viruses are, and always have been, part of diverse social networks, partnerships, households, and family systems, just like anyone else. As social researchers on HIV have argued, an HIV infection “radiates across the entire family system,” and thus families, in whatever form they might take, live with the virus, “not just individuals” ([12, pages: 984, 978], see also [14, 15]). Other authors have made a similar argument about viral hepatitis [16]. In addition, several members and generations of a family might be infected with HIV or viral hepatitis, particularly given the transmission routes for hepatitis B and HIV. In the early HIV epidemic, a call was made to frame the virus as a “family infection” ([17, page: 261], [15]). Since then, attention to families has improved in HIV research, but remains relatively limited in terms of its scope and conceptualisation, and it remains a large gap in the literature on viral hepatitis.

The concept of serodiscordance has provided a way for researchers to move beyond the focus on the individual and explore how viral infections are managed in couple relationships. Much of the work on serodiscordance is dominated by an extensive body of public health literature chiefly concerned with HIV transmission risk in the context of sexual practices, prevention strategies, and reproduction among couples with mixed infection status [18–21]. However, smaller but growing cross-cultural social research literature has broadened this lens to explore the local and lived complexities of couples making sense of and managing HIV serodiscordance. In doing so, it reveals how “HIV risk” is contingent and can be superseded by competing risks and relationship priorities, providing valuable insights into the diverse ways cultural meanings and practices around romantic love, illness, gender, sexuality, medicine, reproduction, and so forth shape serodiscordant intimacy and everyday life among couples [22–25].

Likewise, there is a small body of research that presents unique reflections on the social dynamics at play among couples who inject drugs, including couples who are hepatitis C serodiscordant. This literature has been ground-breaking in changing the lens in hepatitis C research to intimate relationality by elucidating how injecting practices are coproduced in romantic partnerships and informed by perceptions of risk within and outside the relationship, gendered ideas of love and trust, and the specific levels of skill and expertise within these relationships [10, 13, 26–29]. To date, there is no comparable literature on couples in relation to hepatitis B.

This couples' literature has laid a critical foundation for capturing the ways people with intimate connections manage mixed infection status together, and for illuminating serodiscordance as a situated and* lived* phenomenon not reducible to transmission risk. The increasing body of work in this area attests to serodiscordance as a robust and useful concept for understanding the relationality of blood-borne viruses. However, the concept is rarely applied to broader relationships, including family networks beyond the couple. This is despite evidence in the literature (especially the HIV literature) that families are affected by blood-borne viruses in multiple ways, not solely because of potential transmissibility, but because of the social stigma often attached to these viral infections, and the roles families can play in relation to a range of experiences and practices around clinical management, disclosure, secrecy, support, and care-giving. Given this, we argue for the importance of redefining serodiscordance to recognise wider family relationships besides couples. We take families to mean the relational networks of “significant others” [30, 31] that individuals define as “family,” which may include partners, parents, children, siblings, and extended family, as well as friends and other members of families of choice, affinity, or intimate connections [32, 33]. Next, we examine how “families” (beyond couples) figure in the available literature on these blood-borne viruses.

## 3. Families Living with HIV

By now, the HIV literature is vast. The bulk of it is characterised by an individualised focus on transmission, treatment, and prevention. Nonetheless, a sizeable body of research suggests that an HIV diagnosis can have profound effects not only on the infected person, but also on their children, parents, and partners. Some of the effects mentioned include poor mental health outcomes and psychosocial adjustment, altered family dynamics, roles and communication, financial distress and poverty, disruptive changes in housing and loss of community, lack of access to care and support services for family members, gendered expectations around care responsibilities, loss and bereavement, and concerns about the future care of children in case of parental death [12, 14, 15, 34–44].

Children and adolescents are a prominent focus in this literature. A growing number of studies in high-resource countries have investigated the impacts of HIV on both perinatally infected children and those with HIV-positive parents, particularly among minority communities in North America. Common concerns revolve around disclosure to children, whether it be the child's or parent's HIV diagnosis, lost developmental opportunities that alter children's life course due to HIV-related poverty, care responsibilities and disrupted education, as well as psychosocial and psychiatric challenges, sexual risk taking, and delinquent behaviour [45–51]. There is also a substantial literature on the experiences and well-being of AIDS orphans and other children affected by HIV in Sub-Saharan Africa [52, 53].

An overarching theme in the literature is HIV-related stigma and its many consequences for families. Bogart and colleagues found that, in response to perceived or felt stigma, “an atmosphere of secrecy surrounded many families living with HIV” ([36, page: 248], see also [15, 34, 45, 54, 55]). Other studies have similarly noted family concerns about disclosure, privacy, and confidentiality, as well as a tendency towards social withdrawal and silence, rendering care and support needs invisible [12, 14, 37, 39, 46]. In addition to dealing with felt stigma from the outside, some families also struggle with their own “irrational fears of contagion” and “internalized negative attitudes about HIV” ([36, page: 250], also [14, 15]), and might blame or reject the infected member for bringing perceived shame to the family [56, 57].

Overall, the relevant HIV literature is substantial, but predominantly focused on negative outcomes in families affected by HIV. There have been few attempts to specifically examine resilience or helpful management strategies in these families or how HIV might bring about positive changes, such as improved communication, mutual support, trust, and emotional closeness [17, 38, 58, 59]. Apart from the studies on children and adolescents, much of this literature traces the effects and challenges of HIV on families in the pretreatment era (before mid-1990s) and in the era of HIV combination therapy (until mid-2000), or in resource-poor settings in Africa and China. There is scant literature on the needs and experiences of families living with HIV in resource-rich settings in the contemporary era of effective antiretroviral treatment and “HIV normalisation.” Much of the HIV literature that touches on family issues tends to do so from the perspective of only one family member, often the infected person, rather than the whole family. There is also an absence of studies on gay families affected by HIV. And, importantly, with rare exceptions [25, see chapter by Smith et al.], there has been no attempt yet to explicitly extend the concept of serodiscordance beyond couples and its narrow focus on transmission risk, as a way to acknowledge a broader range of HIV-negative family members that play a substantial and ongoing part in the lives of people with HIV.

## 4. Families Living with Hepatitis C

As with the bulk of the literature on HIV, the hepatitis C literature is overwhelmingly focused on individuals, with the exception of public health research on nonsexual transmission in family households [60, 61] and social network research that investigates the role of injecting networks in transmission and prevention [62, 63]. This research clearly shows that hepatitis C and serodiscordance affect broader relationships beyond individuals and sexual partnerships; however these relationships are defined almost entirely in terms of risk, obscuring other meaningful connections in the lives of people who inject drugs. There is also a body of social research exploring peer aspects of hepatitis C prevention among people who inject drugs [64–67] and a small and valuable body of work on couples, as described earlier, but the literature rarely focuses on broader family networks living with mixed infection status.

Yet, families are not entirely absent: scattered references can be found particularly in relation to treatment experiences. Direct-acting antiviral drugs that target the hepatitis C virus have only been available since 2014; before then treatment for hepatitis C was a protracted and notoriously difficult process due to sometimes severe physical and neuropsychiatric side effects and with variable success, making decisions whether to undergo hepatitis C treatment far from straightforward [68–70]. Research from high-resource countries has noted that families are often central to these treatment decisions and can act as either barriers or facilitators. For example, concerns about the potential impact of declining health on the family motivate some to take up treatment, while parental responsibilities discourage others [71–74].

Studies on previous regimes observed that the then gruelling, and often unsuccessful, treatment process and its adverse effects, such as extreme fatigue, mood swings, insomnia, and depression, could have considerable impacts on family dynamics by disrupting their everyday lives and requiring members to take on new roles and responsibilities and find ways to adjust to unfamiliar circumstances and interactions. Such changes often strained relationships and at times created conflict in ordinarily stable households, but also had the capacity to deepen family bonds as the challenges brought about by the treatment tested the strength and resilience of their relationships [69, 70, 75]. Unsurprisingly, emotional and practical family support was identified as a key consideration in people's decision to undertake treatment [69, 71, 76]. Of equal importance, as some researchers and clinicians emphasised, was the need to prepare and support families through the treatment process [68, 70, 77], and it remains to be explored whether this will continue into the current “new treatment” era.

Brief mentions of family also surface in research on hepatitis C related stigma. It is likely that, similarly to HIV, negative cultural discourses around infectious diseases, contagion, and the socially discreditable practices of “high risk” populations could have significant emotional and socially isolating effects not only on affected individuals but also their families. However, there is little exploration of this in the literature. But there is some evidence that the stigma attached to hepatitis C and particularly injecting drug use can strain family relationships and that family members are sometimes seen as a source of stigmatisation, resulting in feelings of rejection and alienation, or reluctance to disclose a hepatitis C diagnosis to the family [78–82].

In summary, although families do appear in the hepatitis C literature, they are rarely at the heart of inquiry. Similarly to the HIV literature, conceptualisations of serodiscordance as a family dynamic are nonexistent. Despite this, there is enough material in the literature to suggest that families can be intimately implicated in treatment experiences and in processes of stigma, which warrants far greater attention to whole-family perspectives and a reconsideration of serodiscordance in the context of living with hepatitis C.

## 5. Families Living with Hepatitis B

Most literature on hepatitis B focuses on epidemiology and on the biomedical impacts of the infection. In contrast to HIV, there is a lack of sociological explorations of living with hepatitis B, including the everyday impacts of the virus on family life and intergenerational relationships. This is despite hepatitis B essentially being a disease of the family, given its primary route of transmission being from mother-to-child (in endemic countries, hepatitis B is most commonly transmitted from mother-to-child or in early childhood through exposure to infected children due to inadequate screening and vaccination programs; the virus can also transmit through sexual contact, shared injecting equipment, donor blood, and other health care procedures [83]). When family is noted in the public health literature, it is mostly in relation to reducing their risk of exposure. Yet, little is known about how serodiscordant families manage prevention, even though studies have noted that the most commonly reported concern among people with hepatitis B is transmission to family members [84–86]. This is particularly troubling as several reviews of the literature have identified significant knowledge gaps and poor understanding of transmission and prevention among people living with or at risk of hepatitis B infection [71, 83, 87, 88].

The literature on hepatitis B literacy mainly focuses on migrants in North America (and to a lesser extent Australia) who come from endemic countries primarily in the Asia-Pacific region, such as Korea, China, and Vietnam [83]. People born in this region make up the majority of the population with chronic hepatitis B in the Global North [89]. Studies highlight that perceptions of hepatitis B among these migrant groups are shaped by personal experiences and cultural understandings about the virus that may differ from those in western societies and medicine [71, 90, 91]. Many migrant groups believe that the virus can spread through everyday activities, such as sharing food and drink, which is significant given that communal eating is an important family and social practice in many Asian cultures [71, 83, 86–88, 92]. Such beliefs often persist following migration, as language barriers in the new country can prevent access to biomedical information about transmission and prevention [93].

Although there is little research on how specific cultural understandings might affect the way that families manage serodiscordance, there are some clues in the literature which suggest that the intrafamilial aspect of hepatitis B can produce quite different responses. For example, an Australian study of culturally diverse migrants noted that testing was commonly sought by whole families, because of the higher risk of transmission within families [90]. A study of Chinese-Americans in San Francisco similarly found that hepatitis B was framed as a family matter, with affected participants keen to educate their family members and encourage them to seek testing to protect the health of the family [94]. There are suggestions that the intergenerational and endemic nature of hepatitis B in Asian communities has the capacity to “normalise” the virus in families and decouple it from stigma and shame in ways that differ quite markedly from hepatitis C and HIV [87, 90, 95, 96]. Conversely, other US studies have found that migrant families can discourage testing due to the importance of maintaining “face” [97] or fear of disclosing status to family members [91]. There is undeniably stigma associated with hepatitis B [83, 98], particularly in China where widespread discrimination against people with hepatitis B has devastating effects on individuals and families [99, 100]. However, the extent of the stigma appears to vary between cultural communities and is “primarily related to poor understanding of prevention and transmission, and not linked to concepts of moral deficit that characterise HIV and hepatitis C related stigma and discrimination” [87, page: 2].

Several of the literature reviews cited above highlight the increasing prevalence of hepatitis B infections among migrant groups and the strain this places on health care systems, and thus the need to better understand how affected communities perceive and respond to the infection. The authors nominate multiple issues needing urgent attention, but none mention serodiscordant family dynamics, which is surprising, given its intergenerational dimension. So far, it remains largely unclear how serodiscordance is experienced and managed in families.

## 6. Understanding Family Dynamics in the Context of Chronic Illness

The broader literature on families living with various other chronic illnesses clearly suggests that family processes and relationships are greatly affected in numerous ways when a member is diagnosed or becomes unwell. Eggenberger and colleagues describe illness as a “family affair triggering families to shift their individual and family patterns as they attempt to manage ongoing life with a chronic illness” [101, page: 283]. They have to “cocreate… a new context for living” and for managing relationships both within the family and with the wider world [59, page: 207]. Crucially, researchers in this field have also found that a family's particular health beliefs, coping strategies, communication patterns, and degree of cohesion or conflict have a critical influence on health-related behaviours, well-being, and health outcomes. This influence is not necessarily without complexities: while family support has been shown to be highly significant to people with a chronic illness, involvement by families can be perceived both as a source of comfort and as stressful and interfering. Likewise, being confronted with a health crisis can foster resilience, growth, and emotional closeness in families, but also evasion and resentment [38, 102–105].

In short, there is ample evidence that illnesses concern not only individuals, but families too in multiple ways. Given that one in two families are likely to experience a chronic illness, as Eggenberger and colleagues [101] state, it is important that research seeks to understand family processes in this context so that support services can adequately meet families' needs and interventions can maximise the potential strengths of their influence [104]. While this is recognised in relation to chronic illnesses more broadly, it remains a gap in relation to stigmatised, transmissible viral infections. In the HIV literature from the Global North, families have certainly figured fairly consistently as a source of support for people living with the virus, and though not conceptualised in terms of serodiscordance, this literature has acknowledged the effects it can have on the health and well-being of family members. With viral hepatitis, however, families have not been a focus of research. Although HIV and viral hepatitis are different in crucial respects, they also share key characteristics, foremost among them transmissibility, social stigmatisation, and their disproportionate impact on marginalised communities. Hence it seems reasonable to speculate that at least some of the issues highlighted in the HIV literature would also be at play in families living with hepatitis B and hepatitis C. But this remains to be explored and documented, as well as the role of families in responding to these infections.

While we have primarily focused on high-resource countries here, our argument for greater focus on families living with serodiscordance is perhaps even more relevant to low-income countries in Asia and Africa where people affected by HIV or viral hepatitis typically live in family clusters or extended kinship networks. In China, for example, the family is the central unit in society and thus a member's diagnosis and the associated stigma greatly impact the identity and social interactions of the whole family [57, 106, 107]. Moreover, in many family-oriented societies, affected people are often dependent on their primary networks for support and survival in the absence of adequate health care infrastructure. As Krishna and colleagues [39] report from India, this can cause great strain on families who are resource-poor, yet it has been largely overlooked.

Evans and Thomas [38] caution that the global spread of neoliberal economic models, including reductions in health spending, might further cement the privatisation and invisibility of care provision (mainly by women and children) within the domestic sphere in low-income countries. In the Global North, these developments are likely to be amplified by biomedical advances that are changing perceptions of HIV as a manageable chronic illness and of viral hepatitis as a treatable condition, no longer requiring or entitling people to the same levels of social support provisions and resources, leading to further cut-backs in services. Despite often being hidden, serodiscordant family life is, as Evans and Thomas summarise, profoundly “embedded in wider structural inequalities and power relations” [38, page: 113] that are produced and driven by economic environments, by cultural perceptions of transmissible diseases and those who acquire them, and by cultural and gendered expectations around family obligations and generational contracts.

Clearly, not only do families play a key role in providing support to people with blood-borne viruses, but they themselves “live with” the virus in their own right. Uphold and colleagues describe families and loved ones as “emotionally coinfected” [108, page: 97]. The direct and indirect impact on children, spouses, parents, and kin networks “extends far beyond individual illness,” influencing the family structure, roles and responsibilities, emotional well-being, social support, and economic resources [12, page: 980]. As such, “coinfected” families face their own challenges and needs that might require support or, at least, attention and better understanding within their specific cultural, epidemiological, and economic context. In coming years, we might see an expansion of this “secondary epidemic” [48, page: 150]. Serodiscordant families are likely to increase in numbers, as treatments are becoming more effective and available and people live longer and are able to safely have uninfected children. This underscores the need to adopt a family-focused approach to serodiscordance in relation to HIV and viral hepatitis. Research and interventions grounded in such a paradigm would prioritise making space for the stories of families to be told and heard, to attain deeper insights into their everyday realities and thus increase the opportunity to better support their needs and build on their strengths [12, 57, 59, 77].

Expanding the concept of serodiscordance in this manner opens up new possibilities in future research on families living with blood-borne viruses. It provides a foundation on which to capture the diverse ways different family members manage mixed infection status in their daily lives. It also provides a case for expanding the definition of family, from specific roles or relationships to a wider gamut of relationships defined as meaningful by the research participants themselves in relation to their experience of serodiscordance.

This broadened definition of serodiscordance can have concrete implications for practice. It can prompt a shift in emphasis from the individual within the clinical setting towards an understanding of the familial context in which infections are experienced, managed and often, especially in the case of hepatitis B, transmitted. This approach is more likely to reflect the experiences of people living with blood-borne viruses and, as a result, may produce more effective clinical interventions. For example, in the case of hepatitis B, the focus on the individual creates a disconnect between people's lived experience of hepatitis B and the infection as described or addressed by clinicians in health care contexts. Recognition of the familial nature of hepatitis B infection would support more effective and socially situated approaches to clinical management, particularly in addressing the challenges related to the lifelong monitoring and/or treatment of the infection.

Services can also be more effective if they recognise that families can take all kinds of forms, and that people with blood-borne viruses need to be supported to define and engage (in an evolving fashion) key family members at critical points in their care, including diagnosis, treatment decisions, service attendance, adherence support, and psychosocial support. But also, critically, in an era of far more effective treatment, recognising the role that key family relationships can play in achieving the necessary high levels of adherence will be essential, as will the involvement of families in achieving the global goal of massively reducing the health risks and social stigma attached to these infections.

## 7. Conclusion: Time to Reconceptualise Serodiscordance

Our purpose in this article has been to make a case for extending the concept of serodiscordance beyond couples with mixed infection status to also include wider family networks. Up until now, limiting the definition and usage of serodiscordance to mixed-status couples (or, in rare cases, to members of injecting networks) is a symptom of the dominant paradigm in the study of viral infections, which frames them in epidemiological terms of “risk,” thus reducing mixed-status couples to vectors and recipients of infection. Limiting conceptualisations of serodiscordance to only intimate sexual or injecting relationships implies that mixed serostatus is only significant in relation to the risk of viral transmission, as if that is what living with a blood-borne virus is all about. As long as this remains the dominant public health focus, the implications for families and social networks go unacknowledged [12, page: 980].

Building on insights from the social research literature on mixed-status couples, it is clear that experiences of serodiscordance are much broader than concerns about risk and that viral infections are intensely relational. Likewise, the literature we have examined here suggests that mixed-status families can be entangled in myriad relational processes, such as social stigma, social isolation, treatment decisions, access to support, disclosure to friends, extended family, and community, cultural health beliefs, gender dynamics, and care responsibilities, all of which are grounded in and negotiated through the politics and practices of serodiscordance. Clearly, people with HIV and viral hepatitis are in a serodiscordant relationship not just with their partner (if they have one), but with other family members too and, indeed, most likely with most people in their lives. Our call to expand the concept of serodiscordance is an attempt to shift the focus to this wider serodiscordant world whose recognition is long overdue.
